# Sex Differences in the Effect of Diabetes on Cerebral Glucose Metabolism

**DOI:** 10.3390/biomedicines9111661

**Published:** 2021-11-10

**Authors:** Chun-Yi Wu, Yu-Hsin Lin, Hsin-Hua Hsieh, Jia-Jia Lin, Shin-Lei Peng

**Affiliations:** 1 Department of Biomedical Imaging and Radiological Sciences, National Yang Ming Chiao Tung University, Taipei Branch, Taipei 112304, Taiwan; chunyiwu@ym.edu.tw (C.-Y.W.); alexa851024@gmail.com (H.-H.H.); 2 Department of Pharmacy, National Yang Ming Chiao Tung University, Taipei Branch, Taipei 112304, Taiwan; ylhsin@ym.edu.tw; 3 Department of Medical Research, China Medical University Hospital, China Medical University, Taichung 404333, Taiwan; 4 Center for Advanced Molecular Imaging and Translation, Chang Gung Memorial Hospital, Taoyuan 404333, Taiwan; solar3520@gmail.com; 5 Department of Biomedical Imaging and Radiological Science, China Medical University, Taichung 404332, Taiwan

**Keywords:** positron emission tomography (PET), streptozotocin (STZ), ^18^F-FDG, hyperglycemia, sexual dimorphism, diabetes, cerebral glucose metabolism

## Abstract

The neuroimaging literature indicates that brain structure and function both deteriorate with diabetes, but information on sexual dimorphism in diabetes-related brain alterations is limited. This study aimed to ascertain whether brain metabolism is influenced by sex in an animal model of diabetes. Eleven rats (male, *n* = 5; female, *n* = 6) received a single intraperitoneal injection of 70 mg/kg streptozotocin (STZ) to develop diabetes. Another 11 rats (male, *n* = 5; female, *n* = 6) received the same amount of solvent through a single intraperitoneal injection. Longitudinal positron emission tomography scans were used to assess cerebral glucose metabolism before and 4 weeks after STZ or solvent administration. Before STZ or solvent injections, there was no evidence of sexual dimorphism in cerebral metabolism (*p* > 0.05). Compared with healthy control animals, rats with diabetes had significantly decreased brain metabolism in all brain regions (all *p* < 0.05). In addition, female diabetic rats exhibited further reduction in cerebral metabolism, relative to male diabetic rats (*p* < 0.05). The results of this study may provide some biological evidence, supporting the existence of a sexual dimorphism in diabetes-related complications.

## 1. Introduction

Diabetes is a metabolic disease characterized by poor glycemic control. It has been considered to be at an epidemic level, as there has been a rising prevalence worldwide over the past few decades. At present, more than 400 million people have been diagnosed with diabetes, and more than 70% of the U.S. population over the age of 65 years have uncontrolled glucose levels [1]. Several health complications have been associated with diabetes. Acute complications include a hyperosmolar hyperglycemic state and diabetic ketoacidosis, and long-term complications can cause blood vessel damage [2]. Blood delivers oxygen and nutrients to organs and tissues to maintain normal function. Of all organs, the brain consumes the largest fraction of cardiac output [3]. Therefore, diabetes-related blood vessel damage can have significant harmful effects on brain function. It has been proven that diabetes increases the risk of stroke [4]. Additionally, studies have also suggested an inextricable correlation between a decline in cognitive function and diabetes [5,6]. As there is increasing evidence that diabetes itself is linked to aberrant brain function, it is of great interest to understand the idiosyncratic patterns of diabetes-related alterations in the brain.

Recent neuroimaging techniques have provided a potential platform for a better understanding of the etiology of human diabetes, and the focus of recent research has also shifted toward understanding how diabetes affects brain structure and function through various techniques. A clear link exists between diabetes and global brain volume reduction [7], and the hippocampus is possibly the most vulnerable to reduction, leading to cognitive dysfunction [8]. Further studies also emphasized that diabetes exerts an unfavorable effect on neural activity [9,10], as hyperglycemia is significantly associated with an increase in the formation of reactive oxygen species [11].

An extensive body of neuroimaging literature indicates that brain structure and function both deteriorate with diabetes. However, an interesting aspect that has been less considered in the past but is now gaining attention is sex-related differences in diabetes-associated alterations in brain function [12]. The role that sex plays in the manifestations and physiological consequences of diabetes and how these differ between males and females is not fully understood. However, sexual dimorphism in diabetes-related complications is apparent. For example, women with diabetes have a higher risk of cardiovascular disease than men [13], and after acute myocardial infarction, the mortality rate is higher in women with diabetes than in men [14]. Along the same lines, it is reasonable to expect that brain function related to diabetes may also be sex-dependent and taking sex into consideration in diabetes studies will help to disambiguate the measures and permit a precise biological interpretation.

The goal of this study was to ascertain whether brain metabolism measured by positron emission tomography (PET), which is known to be lower in patients with diabetes [15,16], is influenced by sex in an animal model of diabetes. Animal experiments, such as in rats, allow longitudinal recording before and after the onset of diabetes, which is not ethical or practical in humans. Extending from previous animal diabetes studies based on measurements during a single, discrete post-diabetes period [17,18], the longitudinal experimental design in this study may advance our understanding of the role of molecular imaging in diabetes-related alterations to brain function.

## 2. Materials and Methods

### 2.1. Animal Preparation

Twelve male Sprague–Dawley (SD) rats (7 weeks old, 245–295 g) and 12 female Sprague–Dawley rats (7 weeks old, 180–195 g) were used in this study. The animals were housed under standard conditions with a 12 h light–dark cycle and provided with a standard rodent diet and water ad libitum. All animal experiments were approved by the local Institutional Animal Care and Use Committee (24 January 2018) and were carried out in accordance with approved guidelines (CMUIACUC-2018-073).

All animals were randomly divided into the control (male, *n* = 6; female, *n* = 6) and streptozotocin (STZ)-induced diabetic (male, *n* = 6; female, *n* = 6) groups at 8 weeks of age. STZ (Sigma Chemical Co., St. Louis, MO, USA) was first dissolved in a 0.1 M sodium citrate buffer before injection. Rats in the diabetic group were injected intraperitoneally with a single dose of 70 mg/kg of STZ, which is a pancreatic beta-cell-specific cytotoxin and is widely used to induce experimental type 1 diabetes in rodent models [19,20]. Rats in the control group received the same amount of solvent intraperitoneally. After injection, body weight and non-fasting plasma glucose concentrations were measured weekly. Plasma glucose concentration was measured using a glucometer (Accu-Chek, Basel, Switzerland). Animals with non-fasting plasma glucose concentrations > 250 mg/dL were defined as diabetic rats and used for the study. As the measurement range for the glucometer is 10 to 600 mg/dL, animals with particularly high plasma glucose levels reaching the top limit can only be recorded as 600 mg/dL. Two male animals died during the follow-up period, leaving five male rats in the diabetes and control groups, respectively.

### 2.2. Positron Emission Tomography Experiments and Data Analysis

An animal micro-PET scanner (Mediso, Budapest, Hungary) was used for PET studies. The PET scans were longitudinally performed on all animals before and 4 weeks after STZ or solvent administration. A dose of 1.0–1.1 mCi ^18^F-fluorodeoxyglucose (^18^F-FDG) was used as a radiotracer for mapping brain glucose metabolism and was injected via the tail vein under isoflurane anesthesia. Following the 60 min uptake of ^18^F-FDG, PET imaging was performed for 20 min. All recorded PET images were used for analysis. Heart and breathing rates were recorded during the entire experiment, and body temperature was maintained using a warm water blanket.

PET images were reconstructed using a three-dimensional ordered-subset expectation maximization method in the acquisition workplace. Regional radioactivity concentrations (KBq/c.c) of ^18^F-FDG were estimated based on mean pixel values within the regions of interest (ROIs). To evaluate the spatial distribution of the diabetes effect, ROIs of the striatum, hippocampus, thalamus, hypothalamus, amygdala, insula, somatosensory cortex, and cingulate cortex were delineated using the PMOD image analysis software (version 4.0; PMOD Technologies Ltd., Zurich, Switzerland). Radioactivity uptakes in these brain regions was decay-corrected to the injection time and expressed as the standard uptake value (SUV) by dividing the radioactivity concentration by the whole-body concentration of the injected radioactivity.

### 2.3. Histological Assessments

After the PET scans were completed, each rat was deeply anesthetized and fixed by perfusing with 200–300 fixative (4% paraformaldehyde in 0.1 M PBS, pH 7.4) through the left ventricle of the heart. Cerebral tissues were kept in 10% formalin for at least 1 week. The brains were then dehydrated with graded ethyl alcohol, cleaned in xylene, and embedded in paraffin. Serial cross-sections (5 μm thickness) of the brain were cut with a microtome, dewaxed with xylene, and hydrated with graded ethyl alcohol. The sections were mounted on glass slides and stained with hematoxylin and eosin (H&E). A light microscope was used to examine the histological changes. 

### 2.4. Statistical Analysis

Measurements are summarized as mean and standard deviation. One-way analysis of variance tests, with repeated measures, were performed on the weight and plasma glucose concentration in each group to compare the differences across different time points. Within the group, ^18^F-FDG uptake values before and after STZ/solvent injection were compared using a paired t-test. To assess region-specific differences between the groups, a Student’s t-test was applied to pairs of animal groups for the 8F-FDG uptake. Statistical significance was set at *p* < 0.05.

## 3. Results

**Figure 1:**
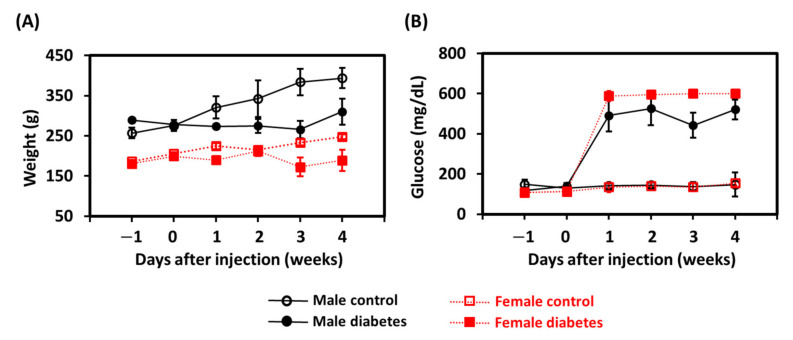
Weekly (**A**) weight and (**B**) plasma glucose level measurements for animals in the control and diabetes groups between sexes.

The weight of and plasma glucose concentrations in the animals are displayed in Figure 1A,B, respectively. Both male and female animals in the control group showed a significant increase in body weight over time (*p* < 0.05), but the plasma glucose concentration did not change significantly after solvent injection. Compared with the age-matched control animals, rats receiving the STZ injection exhibited several diabetic characteristics, including slight weight loss and a significant increase in plasma glucose concentration (*p* < 0.05). Notably, all diabetic females developed glucose values higher than the measurable range (>600 mg/dL), and the plasma glucose could only be recorded as 600 mg/dL. This phenomenon was not observed in the diabetic males, suggesting that females showed more damaged pancreatic beta-cells [21].

**Figure 2:**
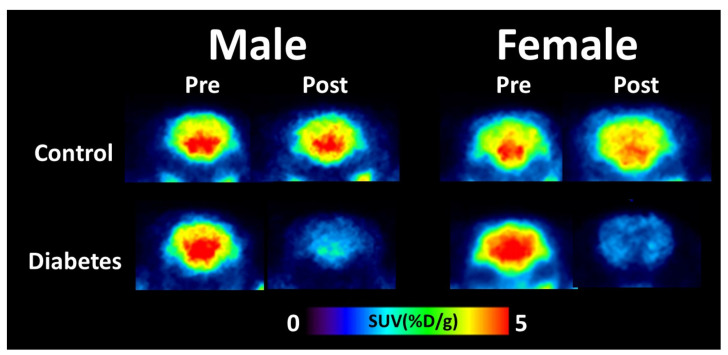
Representative positron emission tomography images of cerebral ^18^F-fluorodeoxyglucose uptake for control and diabetes groups between sexes. SUV, standardized uptake value.

Representative coronal PET images for both the control and diabetic groups are shown in Figure 2. Visual inspection suggested that ^18^F-FDG uptake demonstrated a clear distinction among groups, as the brain uptake of ^18^F-FDG in diabetic rats was lower than that in control animals, regardless of sex.

**Figure 3:**
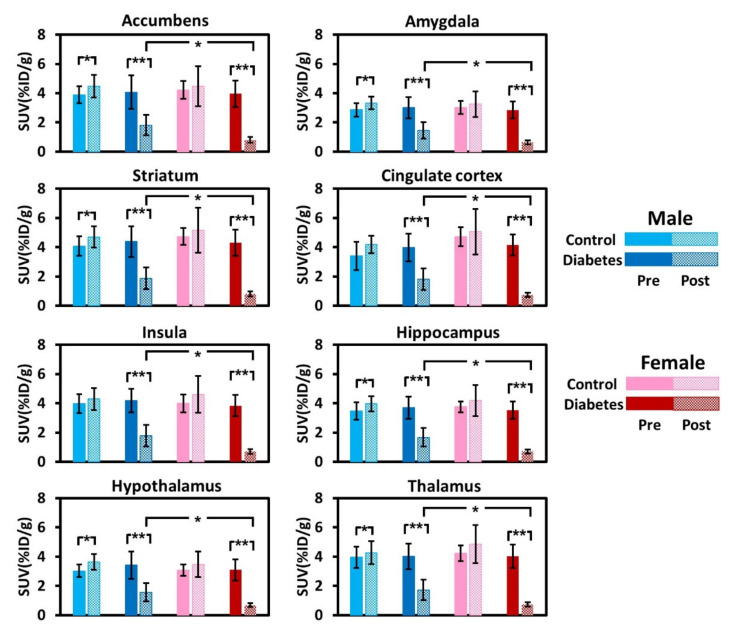
^18^F-fluorodeoxyglucose uptake values of the regions of interest averaged across rats in the control and diabetes groups between sexes. SUV, standardized uptake value. *: *p* < 0.05; **: *p* < 0.01.

Figure 3 plots the group mean values of brain uptake of the ^18^F-FDG before and after STZ or solvent injection among the groups. Before STZ or solvent injection, the patterns of ^18^F-FDG uptake were not significantly different among groups, and there was no evidence of sexual dimorphism in cerebral metabolism. Following STZ treatment, ^18^F-FDG uptake in the diabetic rats was significantly decreased in all brain regions (all *p* < 0.01). In the male animals, ^18^F-FDG uptake values in different brain regions were noted to be 51.4% to 57.4% lower when compared with when they had not received STZ. In the females, ^18^F-FDG uptake values in different brain regions were noted to be 77.6% to 83.9% lower when compared with when they had not received STZ. The degree of reduction in ^18^F-FDG uptake appeared to be sexually dimorphic, with female diabetic rats exhibiting further reductions in cerebral metabolism, relative to the male diabetic rats (*p* < 0.05). A region-specific difference in brain metabolism changes after STZ injection was not detected in both sexes. The magnitude of reduction was homogeneous across different brain regions. In the male control group, ROI analysis further revealed that all brain regions, except the cingulate cortex and insula, showed a significant increase in ^18^F-FDG uptake at 12 weeks of age (all *p* < 0.05), suggesting the normal progression of development. In the female control group, an age-related increase in brain metabolism was not detected (*p* > 0.05). 

**Figure 4:**
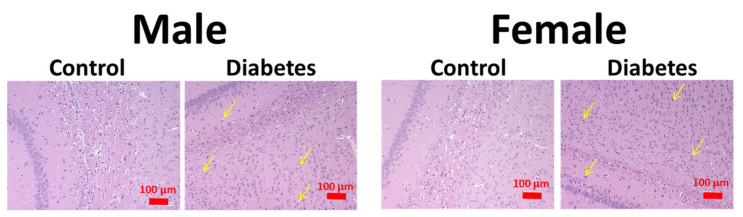
Representative hematoxylin and eosin staining of rat cortexes of different groups. Yellow arrows indicate damaged neurons.

Figure 4 shows a light microscopic examination of H&E-stained sections of the cerebral cortex of the control and diabetic groups. In the control group, a normal distribution of neurons was observed in the cerebral cortex, with smaller glia cells and blood capillaries distributed between neurons. The molecular layer was thick and contained a dense plexus of nerve fibers with few cells. In contrast, the H&E staining revealed morphological changes in the diabetic brain, with neurons appearing as shrunken dark cells. Cells with pyknotic nuclei, condensed cytoplasm, and surrounded halos indicate the cell apoptosis.

## 4. Discussion

The present study demonstrated that diabetes-related alterations in brain physiological function were characterized by a decrease in cerebral metabolism across several brain regions in a type 1 diabetes animal model, and the magnitude of reduction in females was significantly greater than that in males. The results of this study may provide some biological evidence supporting the existence of sexual dimorphism in diabetes-related complications. Unlike previous animal studies, the current experimental design is unique because this is the first study to perform a longitudinal PET scanning before and after the onset of diabetes, providing supplementary information regarding disease progression, without interference from inter-subject variations. 

The primary source of fuel for the brain is glucose, and cerebral glucose metabolism is a valuable index for quantifying brain activity and related neurotransmission. In parallel to this concept, a series of studies have used ^18^F-FDG to track the effect of diabetes on cerebral metabolic activity, and the results concluded that hypometabolism was found in several brain regions in diabetic patients [15,16]. Although the pathogenesis is not fully understood, it is known that diabetes leads to a progressive increase in blood–brain barrier (BBB) permeability, and the disrupted BBB is a critical event leading to the alteration of glucose metabolism [22]. Our results of compromised cerebral glucose metabolism after the onset of diabetes in an animal model are in general agreement with these reports. Notably, glucose utilization was further reduced in female animals with diabetes. Such results are intuitively associated with the fact that female diabetic animals are smaller than their male counterparts at first glance. However, this interpretation cannot account for the similar ^18^F-FDG uptake before the onset of disease between sexes, when the female animals were already smaller. A possible mechanism for the greater decline in the cerebral metabolism of females could be partially attributed to sex-related differences in the inflammatory responses associated with diabetes. C-reactive protein (CRP), a marker of subclinical systemic inflammation, can lead to a compromised BBB and impaired endothelial function. Consequently, a series of immune cascade reactions result in neurodegeneration [23]. CRP is significantly elevated in patients with diabetes, and recent studies found that females with diabetes exhibit higher levels of CRP than males [12,24]. In addition, the diabetic brain relies on ketone bodies as a compensatory fuel to generate adenosine triphosphate [25]. However, sex hormones, such as estrogen, suppress the ketone metabolism system of the brain [26,27]. The accumulative level of ketones negatively impacts the glucose utilization in the brain, and subsequently results in brain glucose hypometabolism [28]. Collectively, this compelling evidence from neurobiological studies supports the assumption that the sexual dimorphism in diabetes-related hypometabolism identified in this study could be considered the real effect.

With respect to control, Zheng et al. showed that diabetic rats had a significant increase in the lactate concentration of the entire brain, except for the cerebellum, using nuclear magnetic resonance spectroscopy [17]. The increased lactate levels could be due to impaired mitochondrial function and turnover [29], which reduce glucose utilization throughout the brain, as demonstrated in this study. These observations are also consistent with a clinical experience showing that dementia tends to have increased lactate levels but decreased cerebral glucose metabolism [30]. Therefore, we speculate that compromised brain metabolism could be a potential mechanism for cognitive decline in patients with diabetes. In particular, the effect of diabetes on incident dementia was found to be greater in women than in men [31,32], which is consistent with the finding of this study, indicating that diabetic female animals showed greater decline in cerebral metabolism.

Our findings of a sex-related difference in the relationship between diabetes and brain function are consistent with a human study that showed that females with diabetes are more likely to be vulnerable to brain damage. Using magnetic resonance imaging (MRI) to measure brain volume, Hempel et al. revealed that brain volume reduction due to diabetes is more prominent in women [12]. However, this specific diabetes-related sexual dimorphism in brain volume reduction was restricted to the hippocampal region, and they failed to detect any sex-associated differences in other brain regions. Meanwhile, Faith et al. showed that elevated glycosylated hemoglobin was associated with lower whole brain volume in females, but not in males [33]. However, the limitation of this study is the lack of regional information. While using molecular imaging in this study, we found that sex-related differences in diabetes-associated brain alterations were generally found in multiple brain regions. As there is no gold standard for comparison, one might argue that data acquired with the PET technique with diabetes-related hypometabolism across several brain regions could occur as a potential overestimation. Combining both structural and functional neuroimaging techniques is a powerful strategy to further investigate how brain dysfunction evolves in patients with diabetes, and one previous study by Last et al. showed that brain volume atrophy due to diabetes was limited in the frontal and temporal regions, while diabetes-associated hypoperfusion affected all regions [34]. As supported by the above study, we tentatively suggest that functional brain changes due to diabetes may occur before structural brain changes and may be more amenable to disease progression. This may also imply that functional techniques were more sensitive in detecting the diabetes-associated brain alterations between sexes. Nevertheless, as structural information was not acquired in this study, employing different approaches to dissect the pathogenic process in the brain may help to shed more light on the impact of diabetes on brain function.

When elderly females lose up to 90% of their premenopausal estrogen levels after menopause, the brain becomes more susceptible to chronic neurodegenerative diseases [35]. As the mean age of the recruited females was about 59 years of age, and most of them were post-menopausal, Hempel et al. hypothesized that the sexual differences in the diabetes-related brain alterations they detected could be partially mediated by the lack of protective effects of estrogen [12]. However, menopause is not a confounder in the sexual dimorphism observed in this study because the female animals used in this study were relatively young adults and had not experienced menopause [36]. Moreover, premenopausal females with diabetes also experience more severe cardiovascular complications than diabetic males [37,38,39]. Collectively, we suggest that sexual dimorphism in diabetes-related brain alterations could be applied to different age ranges, regardless of menopause. To support our hypothesis, including a group of young females with a regular menstrual cycle and a group of elderly females with hormone replacement therapy could further determine the influence of estrogen on diabetes-related alterations between sexes.

As sex plays a role in the manifestation of biological, psychological, and behavioral differences between males and females [40], it is reasonable to expect that brain function should be sex-specific. While this concept is widely accepted, sex differences in brain energy consumption are debatable. By combining non-invasive measurements of cerebral blood flow and venous oxygenation, previous studies have showed that females have higher cerebral oxygen metabolism than males [41,42]. In terms of cerebral glucose metabolism, Baxter et al. found that females tended to have higher values than males [43]. However, some studies have found no evidence of sex-related effects on brain energy consumption [44,45]. In the present study, although brain glucose metabolism differed between sexes after the onset of diabetes, sexual dimorphism in brain glucose metabolism was not apparent in the control groups. Since the sample size was relatively small in this study, we combined control and diabetes groups before STZ or solvent injection as a pooled group to increase the statistical power, resulting in 10 and 12 animals in the male and female subcategories, respectively. The *p* value was 0.35 when comparing whole brain ^18^F-FDG uptake under isoflurane anesthesia between sexes. This *p* value is far from the significant level and increasing the sample size may not significantly improve statistical power. One possibility could be the use of anesthetics, as isoflurane is known to further suppress neural activity [46]. A rodent study by Luft et al. also found that sex-dependent ^18^F-FDG uptake in the brain was only identified in the disease condition, not in the control condition [47]. To further investigate the sex differences in brain energy consumption in animal models, the use of animals under different anesthetics can improve data interpretation.

The major strength of this study is the longitudinal follow-up data, which ensures data compatibility. However, there are several limitations that should be acknowledged. First, both type 1 and type 2 diabetes are chronic diseases which can chronically result in hyperglycemia. However, different types of diabetes may show region-specific metabolic changes and exhibit different patterns of cognitive decline [48,49]. In the case of diabetes-related metabolic changes, an experimental design, similar to the current one but extended to an animal model with type 2 diabetes, is of paramount importance in future studies. Second, to quantify the absolute values of the metabolic rate of glucose, a precise arterial input function (AIF) is needed to determine the cumulative availability of the radiotracer in arterial plasma. However, acquiring an AIF is not practical in an animal model, as invasive catheterization for arterial blood collection may introduce the confounding effect of nociceptive processing on cerebral metabolism during data collection [50]. Therefore, only relative cerebral metabolism quantification was reported in this study and other preclinical PET experiments [46,50]. Third, recent studies have shown that certain gut microbiota play a crucial role in the onset of diabetes [51,52]. While the gut microbiota is closely associated with brain function [53], it may imply that a decrease in cerebral metabolism after the onset of diabetes could be partially attributed to the composition of the gut microbiota. As the present descriptions of the deficits in cerebral glucose metabolism after the onset of diabetes are still in their early stages, analyzing the compositional changes in gut microbiota could provide unambiguous information regarding diabetes-related hypometabolism in the brain. Finally, this study is somewhat limited by its small sample size. In order to make no assumption about the probability distribution of the data set, the non-parametric statistical tests, Wilcoxon rank test, and Mann–Whitney U test, were used to re-evaluate the difference in cerebral glucose metabolism before and after the onset of diabetes, and the comparisons between sexes, respectively. The results showed that the effect of diabetes on cerebral glucose metabolism, and sex-related differences in cerebral glucose metabolism after the onset of diabetes, remain significant (all *p* < 0.05), as when parametric tests were used. Therefore, we believe our conclusions are valid. However, further studies with larger sample sizes could be integrated to allow for more robust statistical analyses.

## 5. Conclusions

The present study provides evidence that brain glucose metabolism decreases with the onset of diabetes. Furthermore, we showed that diabetes-related hypometabolism is sex-specific, with females exhibiting a greater magnitude of reduction relative to males. The characterization of these alterations in brain function may be useful for understanding the mechanisms of diabetes-related changes in cognitive function and the development of sex-dependent intervention strategies.

## Data Availability

The data can be freely given upon request.
